# Prognostic significance of histologic phenotype in periampullary adenocarcinomas

**DOI:** 10.3389/fonc.2024.1407828

**Published:** 2024-07-16

**Authors:** Hee-Sung Kim, Chang-Min Heo, Yoo-Shin Choi, Suk-Won Suh, Seung Eun Lee

**Affiliations:** ^1^ Department of Pathology, Chung-Ang University College of Medicine, Seoul, Republic of Korea; ^2^ Department of Surgery, Chung-Ang University College of Medicine, Seoul, Republic of Korea

**Keywords:** periampullary, carcinoma, prognosis, pancreatobiliary type, intestinal type

## Abstract

**Background:**

Periampullary adenocarcinomas typically exhibit either intestinal or pancreatobiliary (PB) differentiation, and the type of differentiation may be prognostically more important than the anatomic site of origin. This study aimed to evaluate prognostic significance of histological type of periampullary carcinomas.

**Methods:**

Microscopic slides from 110 consecutive pancreatoduodenectomies performed between 2010 and 2020 were reviewed and classified as intestinal or PB type. Clinicopathological factors were compared between PB-(n=93) and intestinal-type (n=17) differentiation.

**Results:**

The intestinal type included significantly more patients with well-differentiated histology (35.3% vs. 11.8%, *p*=0.001) and fewer patients with perineural invasion (41.2% vs. 76.4%, *p*=0.029), advanced T stage (> T3; 41.2% vs.74.2%, *p*=0.007), and systemic recurrence (71.4% vs. 92.9%, *p*=0.005) than PB type. The 5-year-overall survival rate of intestinal-type was significantly higher than that of PB-type (58.8% vs. 20.4%, *p=*0.003). When pancreatic cancer was separately analyzed, the intestinal type showed the best 5-year-overall survival rate, with no significant difference between the PB types excluding PDAC and PDAC (39.4% vs. 19.2%, *p=*0.148). In multivariate analysis, curative resection (hazard ratio, 0.417; 95% CI, 0.219-0.792, *p*=0.008) was the only significant prognostic factor.

**Conclusion:**

Although intestinal histologic phenotype was not an independent prognostic factor on multivariate analysis, it showed pathologic features associated with better survival, while the PB type showed more aggressive tumor biology and consequently worse survival. Further studies are needed to demonstrate the prognostic significance of histologic phenotype.

## 1 Introduction

Periampullary cancer develops around the ampulla of Vater and originates anatomically from the pancreatic head, duodenum, distal bile duct, or ampulla. The World Health Organization (WHO) and American Joint Committee on Cancer (AJCC) have classified periampullary cancer into the above-mentioned four types according to the anatomical location and the tumor, node, metastasis (TNM) staging criteria are different for each origin. (1, 2), However, in the case of the duodenal, distal bile duct, and ampullary cancers, except for pancreatic head cancer which has the worst prognosis, it is often difficult to distinguish the exact site of origin owing to the destruction of normal periampullary anatomy or the presence of epithelial dysplasia even with standardized histopathologic evaluation. Furthermore, this makes it difficult to predict the prognosis and decide on adequate treatment, especially regimen of adjuvant chemotherapy, as the prognosis of patients after pancreaticoduodenectomy (PD) varies greatly even in the same anatomic location (3, 4),. Therefore, several recent studies have questioned this anatomical classification and addressed the need for a new classification (5–7).

Kimura et al. first reported dividing adenocarcinomas originating around the ampulla into intestinal and pancreaticobiliary (PB) types and demonstrated that the latter type had a worse prognosis (8). The intestinal type frequently resembles colon cancer and originates from the intestinal epithelium overlying the ampulla, evolving through an adenoma-dysplasia-adenocarcinoma sequence. In contrast, the PB type originates from the endothelium of the distal common bile duct and distal pancreatic intraepithelial neoplasia in an analogous dysplasia-adenocarcinoma sequence. Although the histologic phenotype in pancreatic ductal adenocarcinoma has not been endorsed by WHO until now, several groups have extended intestinal and pancreatobiliary concepts to the entire spectrum of periampullary adenocarcinoma including pancreatic ductal adenocarcinoma (9–13). In these studies, the intestinal type in pancreatic ductal adenocarcinoma accounted for approximately less than 10% (9–13). Histologic phenotype can be easily determined based on hematoxylin-eosin (H&E) staining. Several studies have shown that the histological type of periampullary adenocarcinomas has biological and prognostic relevance (9–15). However, some other studies have demonstrated no survival difference between the intestinal type and PB type, and the prognostic potential of the histological classification of periampullary cancer has been debated (16–18). These discrepancies between studies may be because of the low incidence of periampullary cancer excluding pancreatic cancer, and the consequent small cohorts with reliable data. Therefore, this study was designed to compare the clinicopathological characteristics and long-term outcomes between intestinal- and PB-type periampullary adenocarcinomas after curative-intent resection in a tertiary referral center to determine the potential of this histological classification as an independent prognostic factor.

## 2 Materials and methods

### 2.1 Study subjects

This study was approved by the Institutional Review Board of Chung-Ang University Hospital, Seoul, Korea. The requirement for written informed consent was waived because of the retrospective nature of the study. We retrospectively reviewed the medical records of patients who underwent curative-intended pancreatoduodenectomy for primary periampullary carcinoma from May 2010 to February 2020 at the Chung-Ang University Hospital, a tertiary referral hospital. Patients who underwent resection with macroscopic residual tumor (R2 resection) or a palliative procedure, and patients with distant metastasis and who diagnosed as other than adenocarcinoma (e.g., adenosquamous cell carcinoma, mucinous adenocarcinoma, and anaplastic type adenocarcinoma) were excluded. In addition, patients with fewer than 6 lymph nodes sampled were also excluded. Finally, a total of 110 patients were included in this study. All PDs were performed by senior pancreatic surgeons. Two expert pancreatic pathologists re-evaluated all pathological slides and determined their histological phenotypes.

### 2.2 Histological phenotype determination

The histological phenotype was determined as intestinal type or PB type based on the criteria first suggested by Kimura et al. and later revised by Saavedra et al. (8), (19), In brief, pancreatobiliary tumors typically feature simple or branching glands and small solid nests of cells surrounded by a desmoplastic stroma. They commonly exhibit cuboidal to low columnar epithelium arranged in a single layer without nuclear pseudostratification. The nuclei within these tumors are typically rounded, although there is notable variation in size and shape from one cell to the next. Intestinal tumors on the other hand, often resembling colon cancer, may present as solid nests with cribriform areas and are characterized by tall and often pseudostratified columnar epithelium. In these tumors, oval nuclei are typically located in the more basal aspects of the cytoplasm, and may also often contain mucin. Cases of mixed-type differentiation were classified according to the dominant pattern. In case of controversy regarding the histological phenotype, a consensus was reached by a second evaluation of the slides. Furthermore, all pathological slides were retrospectively reclassified according to the American Joint Committee on Cancer 8th edition definition.

The analyzed histopathological features included tumor size, histological grade, TNM stage, perineural invasion, and lymphovascular invasion. The tumor stage was defined according to the American Joint Committee on Cancer 8th edition definition. Perioperative medical records were reviewed for patient demographics, surgery type, follow-up, recurrence, and disease-specific survival. Overall survival was defined as the time from surgery to the last follow-up visit.

### 2.3 Statistical analysis

Continuous data are reported as means ± standard deviation (SDs), while numbers and percentages are used for categorical variables. Categorical variables were examined using the Chi-square test. Continuous variables such as maximum tumor diameter were compared using Mann-Whitney U test. Statistical significance was set at *p* < 0.05. The Kaplan–Meier method was used to calculate the curves for overall survival. Cox regression hazard models were used for multivariate analyses. The results are reported as hazard ratios (HR) with 95% confidence intervals (CI). A two-tailed *p* value <0.05 was considered statistically significant. All statistical analyses were performed using the SPSS version 25 for Windows (IBM Corp., Armonk, NY, USA).

## 3 Results

A total of 110 patients underwent curative-intended PD (R0 or R1 resection) and were pathologically diagnosed with periampullary adenocarcinoma at the Chung-Ang University Hospital between May 2010 and February 2020. The enrolled patients comprised 93 (84.5%) patients with PB type and 17 with intestinal-type differentiation. Of the total, 47 patients had extrahepatic bile duct adenocarcinoma, 40 had pancreatic adenocarcinoma, 17 had ampulla of Vater adenocarcinoma, and six had duodenal adenocarcinoma. The characteristics of each histological group are summarized in **Table 1**. The intestinal type had significantly more well-differentiated histology (35.3% vs. 11.8%, *p*=0.001), significantly less nodal metastasis (29.4% vs.55.9%, *p*=0.022), perineural invasion (41.2% vs. 76.4%, *p*=0.029), lymphovascular invasion (23.5% vs. 36.4%, *p*=0.001), and advanced T stage (more than T3; 41.2% vs.74.2%, *p*=0.007) than the PB type. There were no significant differences in the curative resection, adjuvant treatment rates, and recurrence rates. However, systemic recurrence (92.9% vs. 66.7%, *p*=0.005) was more common in the PB type than in the intestinal type.

**Table 1:** Clinicopathologic features according to histologic phenotype.

	Histologic Phenotype		*p*-value
	Intestinal type (n=17)	Pancreatobiliary type (n=93)	
Age, median, y	64.0 ± 9.4	64.0 ± 9.1	0.984
Gender (M:F)	1.5:1	1.7:1	0.507
Tumor origin			<0.001
CBD	8 (47.1%)	39 (41.9%)	
Pancreas	2 (11.7%)	38 (41.2%)	
AoV	6 (35.3%)	11 (11.8%)	
Duodenum	1 (5.9%)	5 (5.4%)	
Tumor size (cm)	3.7 ± 1.4	3.2 ± 1.7	0.665
Elevation of preoperative serum CEA	2 (14.3%)	22 (27.8%)	0.285
Elevation of preoperative serum CA19-9	12 (70.6%)	64 (68.8%)	0.955
Histologic grade			
Well-differentiated	6 (35.3%)	12 (11.8%)	0.001
T stage			
T1&2: T3&4	1:0.7	1:2.9	0.007
T3&4	7 (41.2%)	69 (74.2%)	0.007
Nodal status (N1&2)	5 (29.4%)	52 (55.9%)	0.022
PNI (Yes)	7 (41.2%)	76 (76.4%)	0.029
LVI (Yes)	4 (23.5%)	48 (36.4%)	0.001
Curative resection, R0 (Yes)	15 (88.2%)	78 (83.9%)	0.514
Adjuvant treatment (Yes)	13 (76.5%)	70 (75.3%)	0.092
Chemotherapy (Yes)	10 (76.9%)	39 (55.7%)	
Gemcitabine-based	4 (40.0%)	16 (41.0%)	
5-FU-based	2 (20.0%)	18 (46.2%)	
Others	4 (40.0%)	5 (12.8%)	
Chemoradiotherapy (Yes)	1 (7.7%)	23 (32.9%)	
Radiotherapy (Yes)	2 (15.4%)	8 (11.4%)	
Recurrence (Yes)	7 (41.2%)	55 (59.1%)	0.170
Regional	2 (28.6%)	4 (7.1%)	0.005
Systemic	5 (71.4%)	51 (92.9%)	

Comparing intestinal type, PB types excluding pancreatic ductal adenocarcinoma (PDAC) and PDAC, PDAC group had significantly more advance T stage (more than T3; 33.3% vs. 61.8% vs. 92.5%, *p<*0.001), nodal metastasis (33.3% vs. 41.8% vs. 72.5%, *p=*0.004), perineural invasion (46.7% vs.76.4% vs.85.0%, *p=*0.029), lymphovascular invasion (26.7% vs.36.4% vs.70.0%, *p=*0.001) than the other two groups. (**Table 2**) There were no significant differences in curative resection rates, adjuvant chemotherapy and recurrence rates. For recurrence pattern, systemic recurrence was significantly more common in PB type and PDAC than intestinal type.

**Table 2:** Clinicopathologic features according to histologic phenotype.

	Histologic Phenotype			*p*-value
	Intestinal type (n=15)	Pancreatobiliary type (n=55)	*PDAC (n=40)	
Age, median, y	64.0 ± 9.4	64.0 ± 9.1	63.0 ± 8.3	0.984
Gender (M:F)	9:6	1:1	25:15	0.507
Tumor origin				
CBD	8 (17.0%)	39 (83.0%)		<0.001
AoV	6 (35.3%)	11 (64.7%)		
Duodenum	1 (16.7%)	5 (83.3%)		
Tumor size (cm)	3.7 ± 1.4	3.2 ± 1.7	3.1 ± 1.1	0.062
Elevation of preoperative serum CEA	2 (16.7%)	9 (19.6%)	13 (37.1%)	0.149
Elevation of preoperative serum CA19-9	10 (66.7%)	32 (65.3%)	32 (80.0%)	0.288
Histologic grade				
W/D	5 (33.3%)	8 (14.5%)	4 (10.0%)	0.005
T stage				<0.001
T1,2: T3,4	2:1	21:34	3:37	
T3,4	5 (33.3%)	34 (61.8%)	37 (92.5%)	
Nodal status: N1,2	5 (33.3%)	23 (41.8%)	29 (72.5%)	0.004
PNI (Yes)	7 (46.7%)	42 (76.4%)	34 (85.0%)	0.029
LVI (Yes)	4 (26.7%)	20 (36.4%)	28 (70.0%)	0.001
Curative resection, R0 (Yes)	13 (86.7%)	43 (78.2%)	35 (87.5%)	0.450
Adjuvant treatment (Yes)	12 (80.0%)	40 (72.7%)	31 (77.5%)	0.128
Chemotherapy (Yes)	9 (75.0%)	21 (52.5%)	19 (61.3%)	
Gemcitabine-based	3 (33.3%)	10 (47.6%)	9 (47.4%)	
5-FU-based	2 (22.2%)	7 (33.3%)	9 (47.4%)	
Others	4 (44.4%)	4 (19.0%)	1 (5.2%)	
Chemoradiotherapy (Yes)	1 (8.3%)	12 (30.0%)	11 (35.5%)	
Radiotherapy (Yes)	2 (16.6%)	7 (17.5%)	1 (3.2%)	
Recurrence (Yes)	6 (40.0%)	32 (58.2%)	24 (60.0%)	0.383
Regional	2 (33.3%)	2 (6.3%)	2 (8.0%)	0.049
Systemic	4 (67.7%)	30 (93.7%)	22 (92.0%)	

*, pancreatic ductal adenocarcinoma.

**Figure 1:**
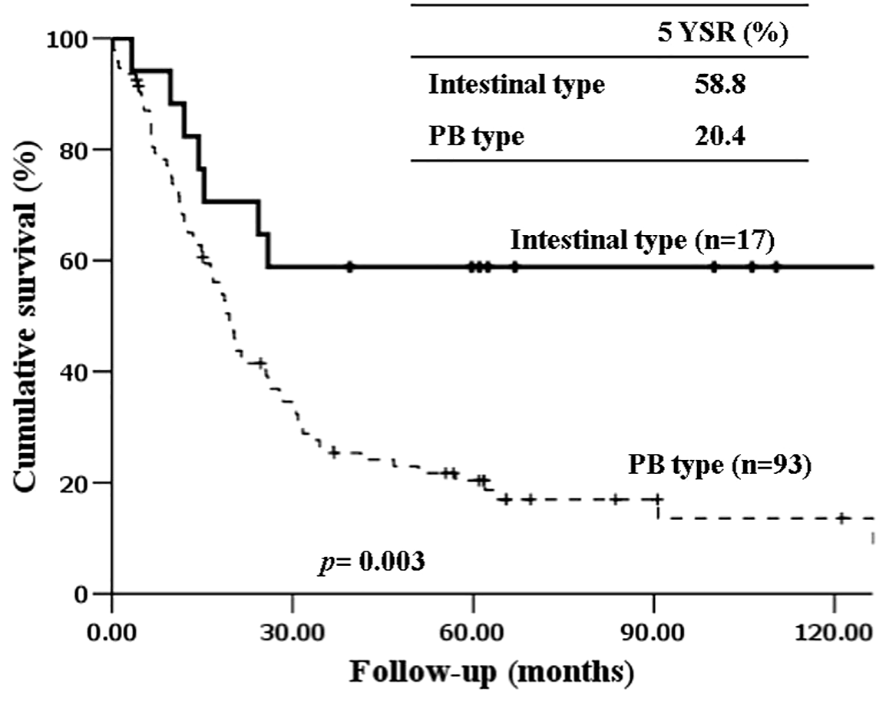
Kaplan-Meier survival curves comparing the overall survival of patients after resection of periampullary adenocarcinomas grouped by histopathologic phenotype.

**Figure 2:**
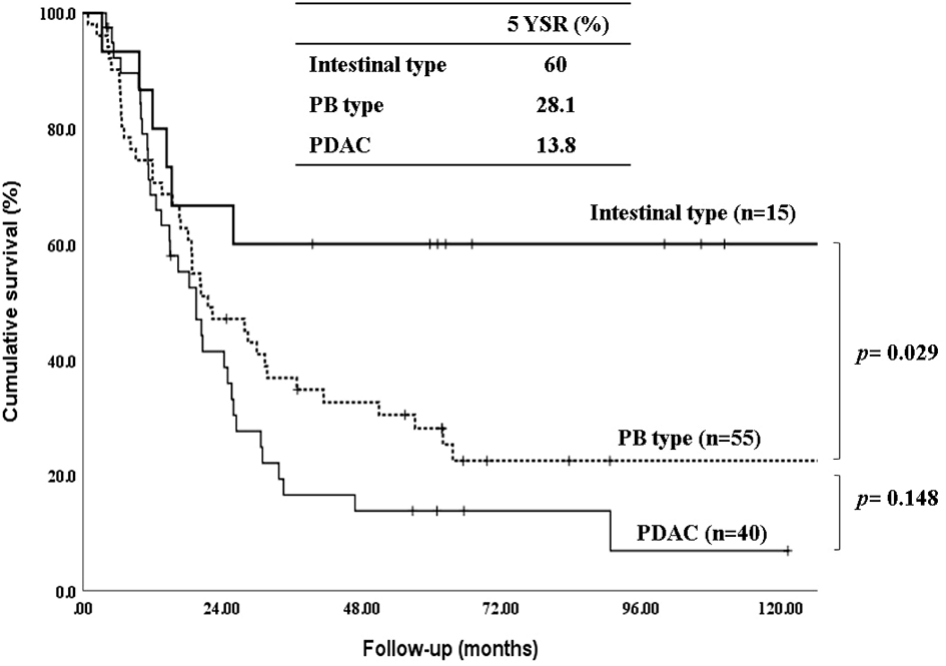
Kaplan-Meier survival curves comparing the overall survival of patients after resection of periampullary adenocarcinomas grouped by Intestinal type, PB types excluding pancreatic ductal adenocarcinoma and pancreatic ductal adenocarcinoma.

**Figure 3:**
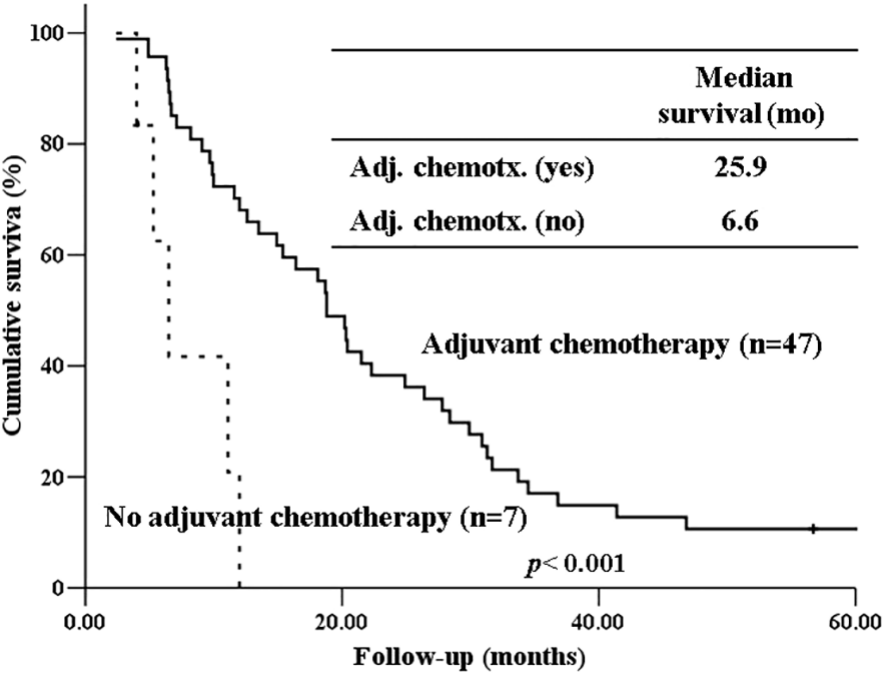
Kaplan-Meier survival curves comparing the overall survival of patients who experienced systemic recurrence depending on whether receiving adjuvant chemotherapy or not.

**Figure 4:**
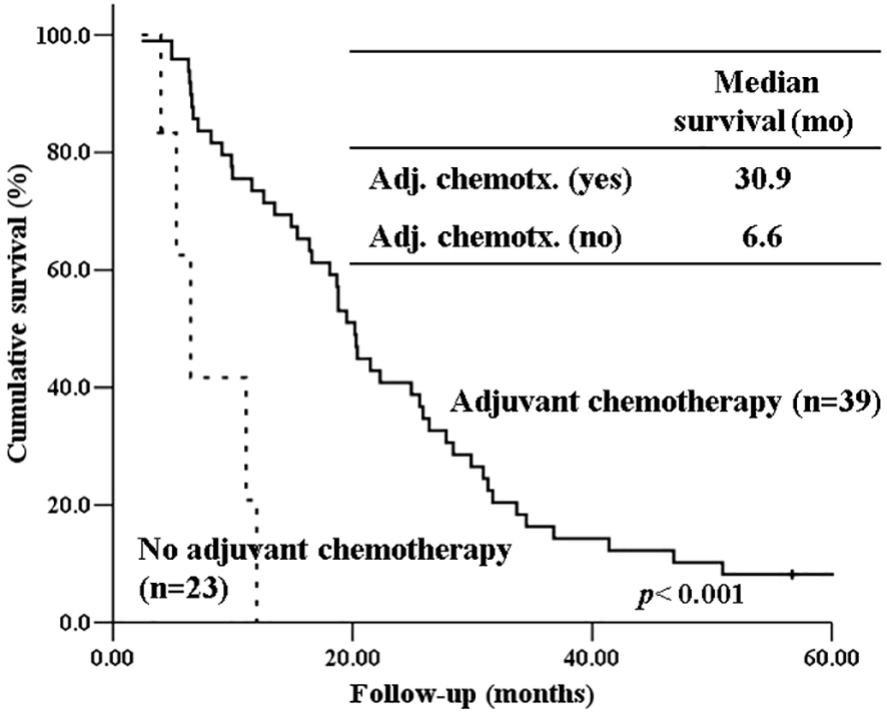
Kaplan-Meier survival curves comparing the overall survival of patients with PB type histology who experienced recurrence depending on whether receiving adjuvant chemotherapy or not.

The 5-year-overall survival rate of the intestinal type was significantly better than that of the PB type (58.8% vs. 20.4%, *p=*0.003) (**Figure 1**), and all patients with intestinal-type ampulla of Vater cancer (n=6) survived for more than 5 years. The 5-year relapse-free survival rate of the intestinal type was also significantly better than that of the PB type (56.3% vs. 17.4%, *p=*0.004). There was no significant difference in the 5-year-overall survival rate between the PB types excluding PDAC and PDAC (39.4% vs. 19.2%, *p=*0.148) (**Figure 2**). There was also no significant difference in the 5-year relapse-free survival rate between the PB types excluding PDAC and PDAC (22.3% vs. 10.9%, *p=*0.289). For patients with systemic recurrence, adjuvant chemotherapy significantly improved survival (median survival, 25.9 mo vs. 6.6 mo, *p*<0.001) (**Figure 3**). However, there was no significant difference between chemotherapy regimens. Furthermore, for patients with PB type with recurrence, adjuvant chemotherapy significantly improved patient survival (median survival, 30.9 mo vs. 6.6 mo, *p*<0.001) (**Figure 4**).

In the univariate analysis, the intestinal type showed a longer mean survival time (MST) than that of the PB type (105.8 months vs. 41.8 months). Non-curative resection (16.8 months vs. 58.2 months, *p=*0.002), presence of lymph node metastasis (39.2 months vs. 64.6 months, *p=*0.018), perineural invasion (37.7 months vs. 97.5 months, *p=*0.003), and lymphovascular invasion (38.9 months vs. 63.8 months, *p=*0.004) showed shorter MST. However, in the multivariate analysis, only curative resection was significantly associated with survival (hazard ratio, 0.417; 95% CI, 0.219-0.792, *p*=0.008). Other factors, such as tumor location, histologic subtype, T stage, N stage, and lymph node metastasis were not significant. (**Table 3**).

**Table 3:** Multivariate Cox proportional hazard regression analysis for histopathologic prognostic factors.

variables	Univariate			Multivariate		
	Subgroup	MST (mo)	*p* value	HR	95% CI	*p* value
Elevation of preoperative serum CEA	Yes	21.2	0.024	1.286	0.585-2.828	0.897
	No	60.0				
Curative resection	R0	58.2	0.002	0.417	0.219-0.792	0.008
	R1,R2	16.8				
Histologic type	I type	105.8	0.003	0.402	0.231-1.801	0.333
	PB type	41.8				
Lymph node metastasis	Yes	39.2	0.018	0.575	0.280-1.180	0.297
	No	64.6				
Perineural invasion	Yes	37.7	0.003	0.545	0.197-1.508	0.519
	No	97.5				
Lymphovascular invasion	Yes	38.9	0.004	0.854	0.425-1.715	0.978
	No	63.8				

## 4 Discussion

Despite the same anatomical location, periampullary cancers show variable prognoses and responses to adjuvant therapy after tumor resection. Histologic subtyping of periampullary cancers into intestinal and PB types is emerging as an alternative to the current classification of anatomic location. This is because histologic subtyping is thought to have the potential to predict the biological characteristics and prognosis of cancer better than the conventional classification based on the anatomical origin of the tumor. Furthermore, some studies have shown that adjuvant systemic therapy improves overall survival in patients with the PB type, mimicking pancreatic ductal adenocarcinoma, whereas the intestinal type does not benefit from the same regimen (20, 21). Therefore, we hypothesized that histopathologic tumor characteristics and long-term survival after curative–intended pancreatoduodenectomies for patients with PB type cancer might be similar to pancreatic ductal adenocarcinoma and have a worse prognosis than the intestinal type.

In the present study, we found that intestinal-type cancers had lower pathological aggressiveness and better prognoses than the PB type. In contrast, the PB type showed clinicopathological features and prognosis similar to those of pancreatic ductal adenocarcinoma. The PB type had significantly more aggressive histology, nodal metastasis, perineural invasion, lymphovascular invasion, advanced T stage, and systemic metastasis than the intestinal type while the PB type and pancreatic ductal adenocarcinoma showed similar aggressive pathological features and poor overall survival. These results further strengthen the consistency of clinicopathological features of histological phenotypes and the possibility of histological classification as a prognostic factor for periampullary cancers rather than anatomic location. A meta-analysis of 37 studies on histological subtype demonstrated similar results to our study (22). PB type had significantly higher rates of advanced T stage, lymph node metastasis, poor tumor differentiation, lymphovascular invasion, perineural invasion, and worse survival than intestinal type. Although most of the studies included in this meta-analysis were focused on ampullary cancer, recently, intestinal type, not only in ampullary cancers but also in pancreatic ductal adenocarcinoma, has shown significantly better survival than PB type (9–12). This had not been demonstrated before due to the relatively rare occurrence of intestinal type in pancreatic ductal adenocarcinoma. Specifically, Westgaard, et al. demonstrated that long-term survival curves of intestinal type of four periampullary tumors, including pancreatic ductal adenocarcinoma, were very similar regardless of tumor location when adjusted for tumor diameter and nodal status (11). Therefore, in cases of periampullary cancer, we suggest determining the histological subtype routinely, in addition to the classification of anatomical location. In most cases, the histological phenotype can be determined by hematoxylin and eosin (H&E) staining alone and may provide more information about adjuvant treatment and prognosis. For morphologically challenging cases, addition of small battery of immunohistochemical markers can improve accuracy (15, 23). A recently published meta-analysis also recommended routine histopathological subtype classification, even in a clinical setting without immunohistochemistry, because the histopathological subtype is a major prognostic factor (22).

Our study also showed that systemic metastasis were more common in the PB type than in the intestinal type. For patients with recurrence, especially those with systemic recurrence, adjuvant chemotherapy significantly improved survival (median survival, 25.9 mo vs. 6.6 mo, *p*<0.001). Furthermore, for patients with PB type with recurrence, adjuvant chemotherapy significantly improved patient survival (median survival, 30.9 mo vs. 6.6 mo, *p*<0.001). Therefore, adjuvant chemotherapy should be considered more actively for PB type cancer. Due to the low number of patients per chemotherapeutic regimen in our study, we were unable to determine if there are differences in patient survival based on specific chemotherapeutic regimens. To date, only a few randomized clinical trials have been performed to demonstrate the role of adjuvant chemotherapy after the resection of periampullary adenocarcinoma, excluding pancreatic ductal adenocarcinoma, because the incidence of each tumor is still low (24, 25).. However, several recent studies have shown that gemcitabine-based adjuvant chemotherapy is associated with prolonged overall survival in the PB type of ampullary cancer but not in the intestinal type (26, 27). There has been no study yet comparing the effects of adjuvant chemotherapy between intestinal type and PB type in pancreatic ductal adenocarcinoma. Future studies should evaluate the validity of stratification by histologic type of periampullary cancers in adjuvant treatment. Therefore, when selecting an adjuvant chemotherapy regimen, the histological subtype should be considered.

An additional issue with histologic type of these cancers is effectiveness of metastatectomy in intestinal type. Given the intestinal type is associated with less aggressive histolopathologic feature, a prior study showed that resection of periampullary adenocarcinoma liver metastasis provides survival benefit only in intestinal type. Considering the benefit of hepatic metastatectomy for the colon cancer and similarity of intestinal type to colon cancer, further study on metastatectomy in intestinal type should be performed near future.

This study had some limitations. First, it was a retrospective, single-center study, leading to a possible selection bias. Second, the sample size may have been too small and unbalance of sample size between PB type and intestinal type may have been too big to show statistical significance or draw definitive conclusions. However, because periampullary cancers are uncommon malignancies and prospective studies are difficult to conduct, a well-organized retrospective study would be a reasonable alternative.

## 5 Conclusions

Although on multivariate analysis, histologic subtype was not an independent prognostic factor because of the small number of included patients, histologic phenotypes, including intestinal and PB types, showed the possibility of being prognostic factors in periampullary cancer. Because of the profound differences in clinical biology and prognosis, the histological classification of periampullary cancer into PB and intestinal types should be encouraged to predict prognosis and choose optimal adjuvant treatment. Further large-scale studies are required to demonstrate the prognostic significance of the histologic phenotype.

## Data availability statement

The data presented in this study are available on request from the corresponding author. The data are not publicly available due to database complexity and local storage guideline.

## Ethics statement

The studies involving humans were approved by Chung-Ang University Hospital Institutional Review Board (IRB No. 1961-003-365). The studies were conducted in accordance with the local legislation and institutional requirements. The ethics committee/institutional review board waived the requirement of written informed consent for participation from the participants or the participants' legal guardians/next of kin because of the retrospective nature of the study and the patients have already passed.

## Author contributions

H-SK: Conceptualization, Data curation, Formal Analysis, Writing – original draft. C-MH: Conceptualization, Data curation, Formal Analysis, Investigation, Methodology, Writing – original draft. Y-SC: Methodology, Software, Validation, Writing – review & editing. S-WS: Methodology, Resources, Software, Visualization, Writing – review & editing. SL: Conceptualization, Formal Analysis, Project administration, Supervision, Validation, Visualization, Writing – original draft, Writing – review & editing.
